# Factors associated with functional incapacity in elders living in long stay institutions in Brazil: a cross-sectional study

**DOI:** 10.1186/1471-2318-14-47

**Published:** 2014-04-15

**Authors:** Inês Echenique Mattos, Cleber Nascimento do Carmo, Lívia Maria Santiago, Laércio Lima Luz

**Affiliations:** 1 National School of Public Health/Oswaldo Cruz Foundation, Rua Leopoldo Bulhões, 1480/817 - Manguinhos, Rio de Janeiro, Brazil; 2 Federal University of Rio de Janeiro/School of Medicine, Rio de Janeiro, Brazil

**Keywords:** Functional dependency, Prevalence, Determinants, Elders, Long-stay institutions for elders

## Abstract

**Background:**

The increase of the elderly population and the high prevalence of chronic diseases have contributed to the increasing importance of functional ability as a global public health problem. This study aimed to assess functional capacity in institutionalized elders, as well as undertake an exploratory analysis of its associated factors.

**Methods:**

This is a cross-sectional study with institutionalized Brazilian elders. Functional capacity was assessed using the Katz Index for Activities of Daily Living (ADL) and the Lawton Scale for Instrumental Activities of Daily Living (IADL). The characteristics of dependent individuals were described and logistic regression models were developed for both scales. Multiple models that included all selected variables were developed using a hierarchical approach. We considered the results from the Wald test (p < 0.05) as a rule for progressing to the next level.

**Results:**

A population of 760 elders was considered. The prevalence of dependence was 50.3% for ADL and 81.2% for IADL. We observed associations between ADL dependence and the following factors: self-report of stroke, difficulty of walking 400 meters, lower total scores in questions related to the temporal orientation section of the cognition test, and self-reports of frequently feeling upset. IADL dependence was associated with educational level, self-report of cancer, difficulty of walking 400 meters, use of glasses, and self-reported memory problems.

**Conclusions:**

Sociodemographic and health conditions were associated with functional incapacity in institutionalized elders. Based on these findings, we emphasize the importance of both prevention and treatment of chronic conditions as well as social support in the maintenance of individuals’ autonomy.

## Background

Functional ability refers to an individual's capacity to carry out the usual activities of any domain of daily life, which are ordinarily undertaken by individuals of the same age and sex [1]. Depending on the conceptual model of functional ability adopted by researchers and the goals of a given study, different measuring instruments have been proposed in the literature to evaluate functional ability. However, the Katz index of Basic Activities of Daily Living (ADL) and the Lawton scale of Instrumental Activities of Daily Living (IADL) are used most often [2,3]. IADL measures the elderly individuals’ ability to be independent and autonomous on eight measures. These include using a telephone, transportation, preparing a meal, managing one's financial resources and own medication, housekeeping, and shopping, among others. ADL comprises less complex self-care activities such as feeding oneself, bathing, walking, and using the toilet [4,5]. In the last few years, there has been a considerable increase in the elderly population and in the prevalence of chronic diseases in this group. Both factors have contributed to making functional ability a relevant issue in public healthcare worldwide.

Previous studies among community-dwelling elders have indicated that age, sex, education, income and occupation, and chronic disease are factors associated with functional incapacity [1,3,6,7]. Other factors related to socio-cultural practices have been shown to have an important role in determining rates of chronic disease and incapacity in community-residing elderly populations [8,9]. Furthermore, the presence of limitations in functional capacity is a predictor of outpatient and hospital services use, of institutionalization and mortality among community-dwelling elders [2]. However, little is known about the role of those factors in the development of incapacity in institutionalized old people.

In Brazil, the demand for care in long-stay institutions for the elderly is growing. Under Brazilian law, the family is the primary source of support and care for the elderly [10]. However, individuals with serious physical and/or cognitive deficiencies and/or social vulnerability generated by insufficient income, by conflicts or by lack of relatives, can be admitted to these institutions [11]. In contrast to other places, these institutions almost always have the character of a permanent residence in Brazil [12]. By law the admission of any elder can be determined according to issues related to their health or to social assistance.

Several studies about the elderly population have been conducted in Brazil, but only a few addressed the functional capacity in institutionalized individuals [13-18].The present study aimed to assess the functional ability of institutionalized elders and to conduct an exploratory analysis of the factors associated with dependence in this population group.

## Methods

We conducted a cross-sectional study from September 2010 to February 2011, with individuals living in long-stay institutions for the elderly in Rio de Janeiro and Juiz de Fora in the Southeast region, and in Campo Grande and Cuiabá in the Midwest region. The Southeast region has a life expectancy at birth of 74.9 years, 5.11% of its population is illiterate, the proportion of people 65 years and over is 8.5%, and its gross national product (GNP) represents 55.4% of the Brazilian total. In contrast, in the Midwest region, life expectancy at birth is 74.3 years, 6.64% of its population is illiterate and the proportion of individuals of 65 years and over is 5.8% while GNP corresponds to only 9.3% of the country’s total [19]. These two different geographical regions were chosen to compare how different sociodemographic, cultural and economic environments influenced the health conditions of this segment of the population [1,8,20,21].

We selected the largest public long-stay institution for elderly individuals in Rio de Janeiro. We also included the largest public institution in each of the other three cities as well as two other institutions, whether public or non-profit, in each. In total the study was carried out at four institutions in the Southeast and six institutions in the Midwest region.

The definition of elder used in this investigation included individuals aged 60 or older, according to the Brazilian Senior Citizen Statute [10]. Individuals eligible for the study's population consisted of those aged 60 or older, living at the selected institutions. We excluded from our study individuals who had been institutionalized for less than 30 days at the date of the interview.

A two-part instrument for data collection was developed for use. The first part consisted of a structured questionnaire including sociodemographic and lifestyle variables, as well as those related to institutionalization, social support networks, and health conditions. The second part contained a set of instruments commonly used in the health assessment of elderly populations: cognition (Mini-Mental State Examination (MMSE); functional capacity (ADL and IADL); and depressive symptoms (15-item Geriatric Depression Scale). With respect to sensory functions, we included questions that inquired about the use of glasses or contact lenses and self-reported vision problems, use of hearing aids, and questions addressing hearing problems. Interviews and health examinations of elderly individuals were conducted by multi-professional teams, duly trained to standardize every procedure. We collected information for all elders who met the eligibility criteria. However, for those who had conditions that made it impossible for them to answer self-reported questions (i.e. dementia, psychiatric disorders, language impairment due to stroke and severe sensory deficits), only sociodemographic variables and information related to mobility and functional capacity were collected from their caregivers.

Katz’s ADL scale allowed us to objectively evaluate six items (bathing, dressing, toileting, transferring to and from a bed or chair, and feeding). A score of 0 or 1 is attributed to each item, depending on how independent the individual is when performing the activity. Individuals who were unable to perform one or more activities without help were considered ADL dependent [21-23].

The Lawton Scale (IADL) was used to assess individual performance in the following activities: using the telephone, using transportation, shopping, taking medications on time, and handling finances. Response categories included: without help, with some help, or cannot do it alone. We considered individuals who were unable to perform one or more activities without help as IADL dependent [4,6,21].

To assess cognition, we used the MMSE, a test consisting of 30 questions (range 0–30 points) in which some dimension of cognition (space and time orientation, memory, calculation, oral and written language) is evaluated. Cutoff points of 18 and 24 reflect the absence or presence of prior formal education respectively, when applied to Brazilian elderly populations [24].

To assess physical mobility, we used questions included in the instrument of the SABE (Health, Welfare and Aging in Latin America and the Caribbean) study [25]. These comprised a set of 12 questions with four possible answers (do with no difficulty, do with some difficulty, do with great difficulty, cannot do) that could be applied to the individual or his/her caregiver. Individuals confined to bed constituted a separate category.

The presence of depressive symptoms was evaluated using the 15-item Geriatric Depression Scale (GDS). A score of five or above was considered indicative of depressive symptoms [26].

Four questions were used to assess social support: religiosity, receiving visitors four or more times per month, self-report of having friends in the institution, and self-report of having friends outside the institution.

The study protocol was approved by the Committee of Ethics in Research of the National School of Public Health (CAAE 0120.0.031.000-10). Informed consent was provided by either the individuals themselves or by a legal representative of the institution if the individual was unable to sign on his or her own.

### Data analysis

In this study, the outcome functional dependence was evaluated by ADL and IADL functional capacity. Descriptive analysis of the distribution of individuals in ADL and IADL dependence groups according to the co-variables in the study was conducted using measures of central tendency and dispersion for continuous variables, and frequency distributions for categorical variables.

For statistical modeling, the variables of interest (ADL and IADL) were categorized in functional capacity and incapacity. The study set out to develop two independent models of factors associated with functional incapacity, one for ADL dependence and the other for IADL dependence. For these two specific analyses, we only included individuals who had had no missing information for the co-variables considered. For ADL analysis, this number consisted of 281 individuals, and for IADL analysis it totaled 424.

We used logistic regression models as a statistical modeling strategy because of the dichotomous dependent variable. Owing to the complexity of the issues and the fact that we used a hierarchical approach for both scales in our analysis, variables were tested in groups or levels, theoretically organized [27] and related to an elder individual's functional capacity in his/her daily life. Groups were organized according to sociodemographic and social support characteristics, health conditions and lifestyle, mobility capacity, and sensory, cognitive, and emotional characteristics.

Each variable was tested using a univariate approach and was selected or not according to the results of the Wald test (p < 0.05) [28]. The crude effect of each explanatory variable on functional capacity was assessed using univariate logistic regression analysis and the odds ratio (OR) was used to estimate risk.

After the preceding step, we proceeded to multivariate analysis, which included only the variables selected through the level approach. The set of sociodemographic variables is considered the least important and it was the first to be tested.

Each successive logistic model included significant variables from the previous models plus new ones. The following configuration was used to test the study variables: model 1 (sociodemographic variables only); model 2 (all significant variables from model 1 plus variables related to family and social support networks); model 3 (all significant variables from the previous model plus those that indicated health conditions and lifestyle); model 4 (all significant variables from model 3 and variables related to mobility assessment); model 5 (significant variables from the previous models plus sensory-motor variables); model 6 (all significant variables from model 5 and cognitive variables) and model 7 (all significant variables from the previous model plus emotional variables).

All analyses were performed using the computer program SPSS (Statistical Package Social Science for Windows) version19.0 (SPSS, Inc., Chicago, IL), 2010.

## Results

The study population consisted of 760 institutionalized elderly individuals, distributed as follows: 254 (33.4%) in Rio de Janeiro, 193 (25.4%) in Juiz de Fora, 153 (20.1%) in Campo Grande and 160 (21.1%) in Cuiabá. The mean age was 76.6 (±9.4), and median was 76 years. Mean institutionalization time was 2.6 (±3.4) years, and median time was 1.4 years. The prevalence of dependence in ADL reached 50.3% (382 elderly individuals) and the prevalence of IADL dependence reached 81.2% (617 elderly individuals).

Table 1 shows the distribution of ADL and IADL dependent individuals according to the co-variables in the study. We identified greater frequency of ADL and IADL dependence among females (58.4% and 51.2%), among those in the oldest age group (48.3% and 42.2%), among illiterate individuals (45.3% and 42.4%), and those who had been institutionalized for less than a year (39.5% and 37.8%). With respect to social support, the ADL dependent group had fewer friends outside the institution compared to the IADL dependent only group (32% vs. 72%). Regarding morbidity, we noticed a high percentage of self-reported high blood pressure (48.4% and 47.3%), diabetes (28.1% and 21.8%), and joint problems (31.4% and 26.4%) in both groups, as well as regular use of more than five medications (31.2% and 28,5%). Weight loss (34.1% and 39,3%), falls (25.6% and 26.4%), and hospitalization in the previous 12 months (20.3% and 19.0%) showed similar percentages in ADL and IADL dependent elders. We noticed that, despite loss of functional capacity, a relatively large percentage of individuals perceived their health as very good or good (37.3% and 41.2%). The number of smokers and alcohol users was greater among those who were IADL dependent (30.4% and 9.0%). We found that 82.2% of ADL dependent individuals and 67.1% of IADL dependent individuals reported severe loss of mobility or being bedridden. High percentages of cognitive deficit (86.5% and 80.2%) and depressive symptoms (57.7% and 52.6%) among elderly individuals with functional incapacity are noteworthy (Table 1).

**Table 1:** Distribution of co-variables among IADL and ADL dependent groups

**VARIABLES**	**ADL (N = 382)**		**IADL (N = 617)**	
	**N**	**%**	**N**	**%**
**SOCIODEMOGRAPHIC**				
**City of the institution**				
Rio de Janeiro	108	28.3	195	31.6
Juiz de Fora	114	29.8	151	24.5
Campo Grande	92	24.1	132	21.4
Cuiabá	68	17.8	139	22.5
**Sex**				
Male	159	41.6	301	48.8
Female	223	58.4	316	51.2
Age group*				
60 to 69 years	67	18.0	131	21.8
70 to 79 years	126	33.8	217	36.0
80 or older	180	48.3	254	42.2
Level of education*				
Illiterate	135	45.3	215	42.4
Incomplete elementary education	89	29.9	179	35.3
Complete elementary education	35	11.7	54	10.7
High school or above	39	13.1	59	11.6
Length of institutionalization*				
Less than 1 year	148	39.5	230	37.8
1 to 3 years	132	35.2	211	34.6
3 or more years	95	25.3	168	27.6
**SOCIAL SUPPORT**				
# Religion*				
Yes	150	93.2	310	92.8
Having visitors*				
Yes	234	62.9	357	59.0
# Friends outside the institution*				
Yes	111	68.5	219	65.4
# Friends inside the institution*				
Yes	122	31.9	244	72.4
**LIFESTYLE**				
# smoking*				
Current smoker	34	21.1	102	30.4
Former smoker	65	40.4	113	33.7
# Alcohol consumption*				
Current consumption	10	6.2	30	9.0
Previous consumption	74	45.7	183	54.8
**HEALTH CONDITIONS**				
# Self-rated health*				
Very good/good	60	37.3	135	41.2
Moderate	59	36.6	119	36.3
Bad/Very bad	42	26.1	74	22.6
# Reported high blood pressure*				
Yes	78	48.4	157	47.3
# Reported diabetes*				
Yes	45	28.1	72	21.8
# Reported cancer*				
Yes	10	6.4	19	5.8
# Reported chronic pulmonary disease*				
Yes	19	11.9	35	10.6
# Reported heart condition*				
Yes	27	17.0	51	15.6
# Reported stroke*				
Yes	49	30.6	68	20.7
# Reported psychiatric problems*				
Yes	31	19.4	60	18.3
# Reported joint problems*				
Yes	50	31.4	88	26.7
Polypharmacy (5 or more medications)*				
Yes	119	31.2	175	28.5
# Reported weight loss*				
Yes	56	34.1	132	39.3
Fall in the last 12 months*				
Yes	97	25.6	162	26.4
Hospitalization in the last 12 months*				
Yes	77	20.3	117	19.0
**MOBILITY**				
Good	30	7.9	104	16.9
Moderate impairment	38	9.9	99	16.0
Severe impairment	240	62.8	340	55.1
Bedridden	74	19.4	74	12.0
**SENSORY**				
Wears glasses or contact lenses*				
Yes	74	27.7	115	24.4
Uses hearing aid*				
Yes	9	3.4	15	3.2
# Reported visual difficulties*				
Yes	44	29.5	81	26.6
# Reported hearing difficulties*				
Yes	38	23.3	61	18.3
**COGNITIVE**				
Cognitive deficit according to MMSE*				
Yes	134	86.5	259	80.2
**EMOTIONAL**				
# Depressive symptoms (GDS)*				
Yes	94	57.7	175	52.6

# Self-reported variables; *Variables with missing data.

Tables 2 and 3 show the final models with both crude and adjusted odds ratios. These correspond to prevalence ratios selected from the individual models with 95% confidence intervals for ADL functional capacity and IADL functional capacity. A strong and statistically significant association between ADL dependence and self-report of stroke was seen (adjusted OR 4.39). ADL incapacity was also associated with difficulty to walk approximately 400 meters (adjusted OR 7.52 for the highest level). The lower the score from MMSE questions assessing temporal orientation, the greater the probability of ADL functional dependence (adjusted OR 4.41 for the lowest level). Regarding the emotional aspects of daily life, we observed that individuals who did not report being frequently upset were less likely to be ADL dependent (adjusted OR 0.21) (Table 2).

**Table 2:** **Explanatory variables**^**a **^**for ADL functional capacity**

**VARIABLES (N = 281)**		**Crude odds ratio**^**b**^	**95% confidence interval**	**Adjusted odds ratio**^**c**^	**95% confidence interval**
**SOCIODEMOGRAPHIC**					
Age group	60 to 69 years	0.36	(0.24 – 0.52)	0.19	(0.06 – 0.66)
	70 to 79 years	0.56	(0.40 – 0.78)	0.19	(0.06 – 0.60)
	80 and over	1.00	-	1.00	-
**HEALTH CONDITIONS AND LIFESTYLE**					
Has a physician ever told you that you had an embolism, cerebral ischemia or thrombosis?	Yes	3.62	(2.21 – 5.93)	4.39	(1.25 – 15.40)
	No	1.00	-	1.00	-
**MOBILITY ASSESSMENT**					
What is the level of difficulty to walk 400 meters?	Impossible/very difficult	15.50	(10.69 – 22.62)	7.52	(2.21 – 25.61)
	Moderate difficulty	3.03	(1.76 – 5.19)	6.56	(2.10 – 20.69)
	Little difficulty/none	1.00	-	1.00	-
**COGNITIVE ASPECTS**					
Temporal orientation	0 and 1	3.70	(2.30 – 5,.5)	4.41	(1.46 – 13.38)
	2 and 3	2.40	(1.43 – 4.02)	1.21	(0.38 – 3.82)
	4 and 5	1.00	-	1.00	-
Reading	Yes	1.00	-	1.00	-
	No	1.49	(1.01 – 2.20)	0.22	(0.07 – 0.63)
**EMOTIONAL ASPECTS**					
Have you been upset lately?	Yes	1.00	-	1.00	-
	No	0.67	(0.46 – 0.99)	0.21	(0.06 – 0.70)

^a^Variables presented in this table were selected in the univariate analysis of each level, according to the results of the Wald test (p < 0.05). Levels were theoretically organized, using a hierarchical approach.

^b^Crude odds ratios represent the crude effect of each explanatory variable on functional capacity assessed through univariate logistic regression analysis.

^c^Adjusted odds ratios represent the effect of each explanatory variable adjusted for all the other variables included in the final model in the multivariate analysis.

**Table 3:** **Explanatory variables**^**a **^**for IADL functional capacity**

**VARIABLES (N = 424)**		**Crude odds ratio**^**b**^	**95% confidence interval**	**Adjusted odds ratio**^**c**^	**95% confidence interval**
**SOCIODEMOGRAPHIC**					
Level of education	Illiterate	3.92	(2.14 – 7.20)	3.32	(1.39 – 7.90)
	Up to 4^th^ grade	1.57	(0.91 – 2.71)	3.56	(1.62 – 7.84)
	5^th^ to 8^th^ grade	0.92	(0.48 – 1.74)	1.52	(0.59 – 3.90)
	Above 8^th^ grade	1.00	-	1.00	-
**HEALTH CONDITIONS AND LIFESTYLE**					
Has any healthcare professional ever told you that you had cancer or a malignant tumor, except for skin tumors (non-melanoma)	Yes	8.07	(1.07 – 60.93)	11.22	(1.15 – 109.13)
	No	1.00	-	1.00	-
**MOBILITY ASSESSMENT**					
What is the level of difficulty to walk 400 meters?	Impossible/very difficult	26.94	(13.40 – 54.17)	39.50	(14.86 – 105.0)
	Moderately difficult	7.67	(3.23 – 18.22)	7.25	(2.81 – 18.71)
	Little difficulty/none	1.00	-	1.00	-
**SENSORY ASPECT: Vision**					
Wears glasses or contact lenses	Yes	0.36	(0.24 – 0.53)	0.45	(0.26 – 0,.7)
	No	1.00	-	1.00	-
**EMOTIONAL ASPECTS**					
Do you often feel abandoned?	Yes	2.45	(1.54 – 3.91)	1.82	(1.01 – 3.27)
	No	1-00	-	1,00	-
Have you been feeling that you have more memory problems than other people your age?	Yes	1.78	(1.04-3.06)	1.93	(0.96–3.91)
	No	1.00	-	1.00	-

^a^Variables presented in this table were selected in the univariate analysis of each level, according to the results of the Wald test (p < 0.05). Levels were theoretically organized, using a hierarchical approach.

^b^Crude odds ratios represent the crude effect of each explanatory variable on functional capacity assessed through univariate logistic regression analysis.

^c^Adjusted odds ratios represent the effect of each explanatory variable adjusted for all the other variables included in the final model in the multivariate analysis.

With respect to IADL dependence, it was found a strong and statistically significant negative association with level of education (adjusted OR 3.32 for the lowest level), with a reduction in dependence as the level increased. We also noticed an association with self-report of having or having had cancer and IADL dependence (adjusted OR 11.22). Difficulty in walking 400 meters was also associated with IADL dependence (adjusted OR 39.50 for the highest level). Wearing glasses or contact lenses had a protective effect against that dependence (adjusted OR 0.45), while self-report of memory problems showed a positive association (adjusted OR 1.93) (Table 3).

## Discussion

The functional capacity of elderly individuals described by ADL and IADL is extremely important for maintenance of their independence and autonomy, and several risk factors associated with incapacity are potentially modifiable [8,29]. The way each individual ages is a result of interactions between biological, physiological, and environmental factors that have influenced his/her life cycle [20]. Reyes-Ortiz et al. [21] studied the functional capacity of elderly individuals in the countries of Latin America and the Caribbean and found a broad variation of IADL and ADL functional dependence. They pointed out that demographic, psychosocial, and socio-cultural variables are mediators in the relationship between health condition and functional ability [21]. A large proportion of Brazil's elderly population has lived in less privileged socioeconomic conditions, and within the current context of our society many of them are institutionalized because of a lack of available caretakers in the family [17].

In this study, we found a high prevalence of ADL and IADL functional dependence ranging from 42% to 62% and 77% to 87%, respectively. These results are similar to those found in other studies with institutionalized Brazilian elderly populations. Araújo and Ceolim [16] evaluated ADL dependence in institutionalized individuals from Taubate, São Paulo, using the same assessment instruments, and found that 63% of those individuals were dependent [16]. A more recent study conducted with 154 institutionalized individuals in Cuiabá, Mato Grosso, found an ADL dependence of 44.2% and an IADL dependence of 83.7% using the Katz and Lawton scales [18]. Figures found in both investigations are similar to those observed in our study. Lee and Choi [30] studied functional incapacity among elder individuals residing in long-term care facilities in Korea and observed that 81% of those living in nursing homes presented ADL dependence compared to 62% of those living in residential care homes [30].

It is important to note that prevalence of IADL dependence was higher than that of ADL in our study and this led to an important difference in the sizes of the samples evaluated in the ADL and the IADL models. Other investigations among community-dwelling or institutionalized elderly populations found similar differences in prevalence of ADL and IADL [4,18,23,30]. Activities evaluated with the Lawson scale are more elaborated, requiring good cognition capacity and a higher level of education, and as so, IADL dependence tends to precede ADL dependence in elders.

In relation to length of institutionalization, a high prevalence of dependence in ADL and IADL was observed among those who had stayed at the institution less than a year. Under Brazilian law, issues related to health or to social assistance can determine the admission of any elderly individual at an institution. Therefore, functional incapacity may be one of the major factors leading to institutionalization in Brazil and recently institutionalized elders would tend to present IADL and ADL dependence. Furthermore, long-term institutionalized elders with poor functional capacity could have already died from chronic health conditions that are conducive to dependence. Those who are still alive would probably be the healthier and, as so, have less functional dependence.

Studies have demonstrated that sociodemographic characteristics have a significant impact on functional capacity levels, even after adjustment for a number of health conditions, among both community-dwelling elderly populations and elders living in long-term care facilities [3,31]. Our findings that advanced age, female sex, and level of education are associated with ADL or IADL dependence are consistent with findings from other studies conducted among community-residing populations both in Brazil [4,6,22,32] and in other countries [8,21,23]. The same findings were observed in studies with institutionalized elders [30,31,33].

Age was an explanatory variable for ADL dependence in our study, maintaining its statistical significance in the final model. The association between advanced age and ADL dependence is consistent with the findings of a few studies conducted with community-dwelling elderly populations, but inconsistent with others [23,34,35]. In relation to institutionalized elders, studies also showed association between advanced age and ADL dependence [30,31,36].

As for IADL dependence, age did not stay in the final explanatory model, similar to a previous study with institutionalized elderly individuals from Cuiaba [18]. On the other hand, a study conducted among a community-residing elderly population found an association between increased age and more IADL dependence [6,23,35].

Reports of difficulty in walking 400 meters in mobility assessments showed a strong association with ADL and IADL dependence. A Japanese study with a community-dwelling elderly population, that also assessed walking difficulties in relation to the incidence of dependence, observed a strong association between the two [23]. Having walking difficulties was related to functional disability in a study conducted in Ontario, Canada, with institutionalized older adults [37]. In our study population, this could be a consequence of problems in the joints, which were highly prevalent among elders, as well as of their relative 'confinement' within the institution. Given that walking is an integral and necessary part of the ADL and the IADL, reported problems in that area may be an early sign of functional dependence [23]. Walking was the most frequent problem reported by Korean elders living in long-term care facilities [30].

Chronic diseases may also contribute to reducing an individual's functional independence as shown in studies among community-residing elderly populations [2,8,38]. One explanatory variable of ADL dependence in our sample was self-report of stroke. This disease was also associated with incapacity among institutionalized elders in Korea [30]. The consistency of these findings with those of other studies of functionality in elders indicates the importance of preventing and treating chronic conditions to maintain the quality of life of elderly individuals [8,30].

Visual impairment was a risk factor for functional incapacity in a study conducted in the United States among community-dwelling elders [2]. In our study, the fact of wearing glasses or contact lenses was a protective factor for IADL incapacity. One possible explanation could be that many institutionalized elders have no access to glasses or lenses and so the individuals who actually wear them could be those receiving adequate care. On the other hand, this could be a chance finding since we estimated associations between a series of co-variables and IADL in the present study. A longitudinal study conducted in Canada found that vision at baseline was a predictor of ADL incapacity in institutionalized elders [37].

Several studies have pointed out the association between cognitive deficits and functional incapacity among community-residing elderly populations [23,39,40]. In another study with Brazilian institutionalized elderly individuals, those who had cognitive impairment presented more functional incapacity than those who had not [18]. In this study, poor temporal orientation and difficulty in reading, both items of the MMSE, were explanatory variables of ADL dependence. Studies with community-dwelling elderly individuals also showed an association between IADL and/or ADL functional incapacity and cognitive deficit, as well as with an individual's level of education [2,6,40]. Analyzing cognitive functioning and functional disability in home care elders in Michigan, Li and Conwell observed an association with both ADL and IADL dependence [41]. This suggests that intellectual activity and good cognitive function are important conditions for healthy aging.

It has been suggested that physical and social activities and a feeling of fulfillment with respect to life may operate as barriers against the negative impact of chronic health conditions in elderly individuals, although such inter-relationships are not yet well understood among community-residing elderly individuals [38]. Social relationships tend to become more limited with advanced age, and especially in the case of institutionalized elders they are greatly limited.

Emotional aspects, assessed by the 15-item GDS, were explanatory variables of ADL and IADL dependence in this study. In a study conducted with community-dwelling Japanese and South Korean elders, high scores in a scale that assessed depressive symptoms were associated with decreased functional ability [39]. In a study with American institutionalized elders, the authors also found associations between depressive symptoms and ADL and IADL dependence [18].

Initial data from a Brazilian study indicated the existence of demographic, economic, social, and cultural differences among institutionalized elders in the Southeast and Midwest regions [42]. Therefore, differences related to functional ability were initially expected. However, in the final model, there were no significant differences between them. Nevertheless, owing to the relevance of sociodemographic variables in functional capacity assessment, we recommend considering their distribution when making comparisons with results from studies conducted in different urban contexts. Furthermore, one should analyze the findings of this study with caution, since it consists of a cross-sectional design, and as such, it does not offer the possibility of identifying which variables, among all those included, preceded the others.

Maintaining functional independence in old age is a complex phenomenon that has only been partly explained by the variables collected in this study. However, the consistency of our findings and the strength of some of the observed associations points to a number of possible interventions that could prevent the elderly population from developing functional dependency. Further studies would be necessary to expand our knowledge about the impact of health conditions and social inequalities on elderly individuals' functional capacity especially with respect to social and financial resources, self-esteem, and life fulfillment.

## Conclusions

This study provides estimates of functional incapacity in ADL and IADL for institutionalized Brazilian elders. It shows a high prevalence of dependence in the types of activities measured by both scales. As an exploratory analysis of factors associated with these conditions, this study included a large set of variables related to the capacity to function in elders. Our results are consistent with previous findings, and show that sociodemographic variables such as age, gender and educational level, health conditions, mobility, cognitive, and emotional characteristics are associated with functional incapacity.

Based on these findings, some recommendations can be made for the maintenance of functional ability in institutionalized individuals. The prevention and treatment of chronic conditions and a healthier lifestyle would promote autonomy and a better quality of life. Social support from family and institutional staff, and involvement of the elderly in social activities would help diminish negative life attitudes and contribute to the prevention of cognitive impairment. Finally, health education activities should be conducted regularly in the institutions to improve the quality of the care provided for those who live there.

## Abbreviations

IADL: Instrumental activities of daily living; ADL: Activities of daily living; MMSE: Mini-mental state examination; OR: Odds ratio; GNP: Gross national product.

## Competing interests

The authors declare that they have no competing interests.

## Authors’ contributions

IEM and LMS conceived of the study, participated in its design and coordination and collaborated in the statistical analysis. LLL participated in the coordination of the study and in its design. CNC participated in the design of the study and performed the statistical analysis. All authors helped to draft the manuscript and read and approved its final version.

## Pre-publication history

The pre-publication history for this paper can be accessed here:

http://www.biomedcentral.com/1471-2318/14/47/prepub
